# Characterization of culture-positive adenovirus serotypes from respiratory specimens in Toronto, Ontario, Canada: September 2007–June 2008

**DOI:** 10.1186/1743-422X-6-11

**Published:** 2009-01-26

**Authors:** Rani Yeung, AliReza Eshaghi, Ernesto Lombos, Joanne Blair, Tony Mazzulli, Laura Burton, Steven J Drews

**Affiliations:** 1 Ministry of Health and Long-Term Care, Public Health Laboratories Branch, Etobicoke, Ontario, Canada; 2 Department of Microbiology, Mount Sinai Hospital, Toronto, Ontario, Canada; 3 Department of Laboratory Medicine and Pathobiology, University of Toronto, Toronto, Ontario, Canada

## Abstract

This study describes the prevalence of culture-positive adenovirus serotypes in culture-positive respiratory specimens sent to the Central Public Health Laboratory, Toronto, Ontario, Canada for the period September 2007–June 2008. Total nucleic acid was extracted from virus cultures using an automated extraction method followed by polymerase chain reaction and Sanger sequencing of the adenovirus hexon gene hypervariable region 7. 73% of specimens (n = 70) were from patients ≤ 4 years of age. Of the 96 adenovirus isolates, the most common identified serotypes were serotype 3 (n = 44, 46%), serotype 2 (n = 25, 26%), serotype 1 (n = 17, 18%), and serotype 21 (n = 5, 5%). Adenovirus serotype 14 was not found in this study group. The leading serotype, Ad3, was identified throughout the duration of the study period. Molecular methods allow for the determination of circulating adenovirus serotypes and be used to document the spread of highly virulent adenoviral serotypes into a region.

## Findings

Adenovirus causes a range of diseases including respiratory tract infections, conjunctivitis, and gastroenteritis. Recent respiratory outbreaks of a virulent strain of human adenovirus 14 (Ad14) led to the deaths of nine people in the United States during the 2006–2007 respiratory season [1]. As part of a respiratory viral pathogens initiative, the Central Public Health Laboratory initiated a surveillance program for the detection and typing of culture-positive adenovirus serotypes isolated from respiratory specimens.

This program utilized molecular protocols previously designed for the serotyping of adenoviruses [2]. The purpose of this study was to determine the prevalence of culture-positive adenovirus serotypes in the Greater Toronto Area during the 2007–2008 respiratory season and to determine whether adenovirus 14 was circulating in this region.

The Central Public Health Laboratory is the primary virology diagnostics and reference facility for the Greater Toronto Area, covering a population of 5.5 million (2006 census data). The study included all adenovirus isolates detected in culture that were linked to a respiratory specimen collected between September 1, 2007 and June 19, 2008.

Forty-two adenovirus control strains were purchased from the American Type Culture Collection and the National Institute of Health. Briefly, specimens were cultured on rhesus monkey kidney cells (Diagnostic Hybrids, Athens, OH, USA) in atmospheric air for 10 days followed by post-culture direct fluorescence antibody staining for adenovirus (Diagnostic Hybrids, Athens, OH, USA).

Total nucleic acid was extracted from positive cultures following the off-board lysis protocol with the NucliSENS easyMAG (bioMérieux). The extractions yielded 55 μL of total nucleic acid per sample, which were subsequently aliquoted by 5 μL amounts into PCR strip tubes to avoid contamination of the extracted stocks of DNA.

Conserved segments surrounding the HVR-7 of the hexon gene were targeted by primers described by Sarantis et al [2]. 5 μL of amplicon was loaded with 1 μL of 6× Loading Dye (Fermentas, Burlington, ON, Canada), and the Low Range MassRuler DNA Ladder (Fermentas, Burlington, ON, Canada) was used to estimate the concentration of DNA in each sample. Both strands of the amplified DNA were labeled using the BigDye Terminator v.3.1 Sequencing Kit (Applied Biosystems, Foster City, CA, USA). Each sequencing reaction contained 2 μL BigDye Sequencing RR-100, 3 μL Sequencing Buffer, 2 pmol of forward or reverse primer, 10 ng of DNA, and PCR-grade water to a total reaction volume of 20 μL. The labeling was carried out by a BioRad iCycler with an initial denaturation step of 96°C for 1 min, 25 cycles of denaturation at 96°C for 10 s, annealing at 55°C for 5 s, and elongation at 60°C for 4 min. Unincorporated BigDye was removed using the DyeEx 2.0 Spin Kit (Qiagen, Mississauga, ON, Canada).

All samples were sequenced using the ABI Prism 3100 Genetic Analyzer (Applied Biosystems). Sequence editing and alignment were completed with FinchTV and ClustalX 2.0 for Windows. BLAST results were ranked by score and identity.

The Chi-squared test was used to estimate distributions of adenovirus in patient subpopulations GraphPad Prism 5 (GraphPad Software, Inc. La Jolla, CA, USA) and values of p < 0.05 were considered to be significant.

As none of the respiratory specimens were from known respiratory outbreaks, all were considered sporadic cases of respiratory illness. Patient characteristics are identified in Table 1. 73% (70/96) of adenovirus isolates were from patients who were ≤ 4 years of age. As shown in Table 1, the most common adenovirus serotypes were Ad3 (46%, n = 44/96), Ad2 (26%, 25/96), Ad1 (18%, 17/96) and Ad21 (5%, 5/96). No Ad14 were identified among the remaining isolates. The distribution of the four most common serotypes Ad3, Ad21, Ad2, and Ad1 between patients ≤ 4 years and >4 years of age was significantly different p < 0.0001, χ^2 ^= 24.43, df = 3). Patients ≤ 4 years of age had the serotype distribution; Ad3 (37%, n=26/70), Ad2 (36%, n=25/70), Ad1 (21%, 15/70), and Ad21 (1%, 1/70). Patients >4 years of age had the serotype distribution; Ad3 (71%, n=17/24), Ad2 (0, n=0/24), Ad1 (4%, 1/24), and Ad21 (17%, 4/24). An analysis of the temporal distribution of serotypes identified an equitable distribution of the major adenovirus serotype (Ad3) over all months of the collection period.

**Table 1:** Serotype and age distribution of clinical isolates

			**Age Distribution (n)**	
**Subgroup**	**Serotype**	**No. (%) of Isolates**	0–4 yrs.	>4 yrs
B	3	44*(46%)	26	17
	21	5 (5%)	1	4
C	1	17* (18%)	15	1
	2	25 (26%)	25	0
	5	1 (1%)	1	0
	6	1 (1%)	1	0
D	8	1 (1%)	0	1
	45	1 (1%)	0	1
E	4	1 (1%)	1	0

*Age of one patient undetermined

The majority of isolates (93/96, 97%) were from subgroups B (serotypes 3,7,11,14,16,21,34, an 35) and C (serotypes 1,2,5, and 6) which have been identified as respiratory pathogens in other previous studies [3-5]. From these subgroups, Ad3, Ad2 and Ad1 were identified as the most common serotypes in this study population as a whole and within the subsets of patients ≤ 4 years of age. It should be noted that the majority (70/96, 73%) of specimens were from patients ≤ 4 years of age. This data is consistent with recent surveys of adenovirus serotypes in pediatric populations which have identified Ad1, Ad2, and Ad3 as the most common circulating serotypes in pediatric patients with acute respiratory disease [6,7]. The predominance of Ad3 in the adult population has also been described by previous authors in other geographic regions [8].

The authors note that Ad14 was not identified in this study. This data is comparable to a recent pediatric survey from Texas which also failed to identify Ad14 despite previous reports of Ad14 in that region[7]. Acute respiratory disease associated with Ad14 has been identified in New York, Washington, Oregon and Texas [1] and the geographic location of our province adjacent to New York State was suggestive that Ad14 might be circulating in Ontario. Although no Ad14 was identified in the Greater Toronto area, the authors note that this study does not preclude the possibility that Ad14 or other novel genotypes were circulating in other regions of the province. Future studies over longer-time periods that rely on PCR for the detection of respiratory adenoviruses may be able to provide a more comprehensive understanding of circulating adenovirus serotypes [9].

In conclusion, this study indicates that Ad3, Ad2, and Ad1 were the most common adenovirus serotypes circulating in the Greater Toronto Area between September 1, 2007 and June 19, 2008. Ad14 was not identified in this patient population during the period of analysis. This comprehensive means of typing adenovirus allow public health laboratories to inform their stakeholders on the possibility of new emergent strains which in turn should allow the stakeholders to undertake appropriate infection control and public health steps when these strains arrive in a region. It is evident from this study that sequence-based methodologies for the typing of adenovirus from viral culture materials are an effective means of determining adenovirus serotypes in specific communities and populations.

## Abbreviations

Ad: adenovirus; Ad1: adenovirus serotype 1; Ad2: Adenovirus serotype 2; Ad3: adenovirus serotype 3; Ad14: adenovirus serotype 14; HVR-7: hypervaiable region 7.

## Competing interests

The authors declare that they have no competing interests.

## Authors' contributions

RY undertook experiments and wrote initial manuscript, AE assisted in sequencing protocols, EL assisted in PCR optimization, and sequencing protocols, JB assisted in experiment planning and resource acquisition, TM provided clinical specimens, LB undertook adenovirus culture detection, SJD planned, coordinated, and supervised project and helped write manuscript.

## Authors' information

Rani Yeung is an undergraduate student at the University of Toronto. Ms. Yeung undertook this work as a Summer project supported by the Ontario Government Summer Experience Program.
